# Turn on, Tune in, and Drop out: Predictors of Attrition in a Prospective Observational Cohort Study on Psychedelic Use

**DOI:** 10.2196/25973

**Published:** 2021-07-28

**Authors:** Sebastian Hübner, Eline Haijen, Mendel Kaelen, Robin Lester Carhart-Harris, Hannes Kettner

**Affiliations:** 1 Centre for Psychedelic Research Imperial College London London United Kingdom

**Keywords:** attrition, digital data, dropout, educational level, personality, psychedelics, web-based research, web-based survey

## Abstract

**Background:**

The resurgence of research and public interest in the positive psychological effects of psychedelics, together with advancements in digital data collection techniques, have brought forth a new type of research design, which involves prospectively gathering large-scale naturalistic data from psychedelic users; that is, before and after the use of a psychedelic compound. A methodological limitation of such studies is their high attrition rate, particularly owing to participants who stop responding after initial study enrollment. Importantly, study dropout can introduce systematic biases that may affect the interpretability of results.

**Objective:**

Based on a previously collected sample (baseline n=654), here we investigated potential determinants of study attrition in web-based prospective studies on psychedelic use.

**Methods:**

Logistic regression models were used to examine demographic, psychological trait and state, and psychedelic-specific predictors of dropout. Predictors were assessed 1 week before, 1 day after, and 2 weeks after psychedelic use, with attrition being defined as noncompletion of the key endpoint 4 weeks post experience.

**Results:**

Predictors of attrition were found among demographic variables including age (β=0.024; *P*=.007) and educational levels, as well as personality traits, specifically conscientiousness (β=–0.079; *P*=.02) and extraversion (β=0.082; *P*=.01). Contrary to prior hypotheses, neither baseline attitudes toward psychedelics nor the intensity of acute challenging experiences were predictive of dropout.

**Conclusions:**

The baseline predictors of attrition identified here are consistent with those reported in longitudinal studies in other scientific disciplines, suggesting their transdisciplinary relevance. Moreover, the lack of an association between attrition and psychedelic advocacy or negative drug experiences in our sample contextualizes concerns about problematic biases in these and related data.

## Introduction

Psychedelic substances, such as mescaline, psilocybin, or dimethyltryptamine, have likely been consumed by humans for thousands of years through different species of plant and fungi [1,2]. After a period of promising scientific exploration, especially of lysergic acid diethylamide as a psychiatric treatment aid in the 1950s and 1960s, classical psychedelics, defined here as psychoactive compounds eliciting their effects on cognition and perception through agonistic action at the serotonin 2A receptor, have been policed restrictively in most Western countries, largely prohibiting academic research [3]. The ongoing resurgence of studies into the psychological and neural effects of psychedelic substances, sometimes referred to as a “psychedelic renaissance” [4], is now being paralleled by commercial and policy developments, reflecting a progressive social acceptance of psychedelic substances both as medicinal tools in the treatment of addictive and mood disorders [5] and for psychological benefits in healthy subjects [6].

The increasingly widespread use of psychedelics [7,8] together with advancements in digital data collection techniques, have motivated the development of a new type of ecological study focused on gathering large-scale longitudinal data sets from psychedelic users by using prospective study designs; that is, before and after the naturalistic use of a psychedelic compound. This approach has already yielded improved, ecologically valid models of the notoriously difficult-to-predict psychedelic state and its outcomes [9-11]. Efficient recruitment and data collection, low cost, and avoidance of human transcription errors represent relevant advantages of web-based survey studies such as these. However, there are also significant limitations, including the lack of experimental controls, participant accountability, and data validity, which may weaken inferences that can be drawn from the data [12,13]. Study attrition and self-selection are particular issues that could skew study samples in potentially problematic ways; for example, by promoting confirmation biases that exaggerate or downplay risks or benefits [14]. Such biases may be especially poignant when interventions that have a special value or significance for the participant are assessed [15]. Accordingly, researchers are obliged to place caveats on data collected via web-based surveys, emphasizing their preliminary, nonconfirmatory nature.

Previous studies have indicated that younger age [16-19], lower educational levels [20-26], and unemployment [24,27,28] are among the most reliable predictors of premature termination of studies or discontinuation of treatment compliance in several contexts. Psychological variables associated with poor compliance and retention include poor mental health [29-34] and low conscientiousness scores [35,36], and there is some evidence that high extraversion [37] and low agreeableness [1,38] might also be risk factors for dropout.

Although the issue of attrition in psychedelic research has not yet been addressed empirically, it has been discussed previously [9,10,39] as a significant limitation in observational psychedelic survey studies, potentially affecting the interpretability of results owing to psychedelic-specific variables associated both with psychological outcomes and the likelihood of dropout. Specifically, positive biases toward psychedelic substances have been identified in previous opportunity samples [9,40] and have in 1 case been shown to predict the increase in self-reported psychological well-being following psychedelic use [9]. A positive relationship between participant bias and the likelihood of study completion could thus represent a potential confounder leading to biased outcomes. Similarly, it is conceivable how particularly unpleasant or difficult psychedelic experiences, which are known to negatively impact long-term psychological outcomes [11], could reduce the motivation of participants to continue responding to a study, thereby creating a systematic attrition bias specific to prospective studies on psychedelics.

In this study, we used data from a published prospective assessment of the effects of psychedelic drug use on various subjective psychometric outcomes [9]. This particular study focused on identifying response predictors. The primary outcome was prospective change in psychological well-being, and consistent with a prior controlled study [41] the quality of the acute psychedelic experience was a strong predictor of longer-term outcomes, where, for instance, positive “mystical-type” experiences and high “emotional breakthrough” scores [11] were significantly predictive of improvements in psychological well-being. Problematically, however, attrition rates in this study were high, with only 29% of the total sample completing surveys up to the primary endpoint 4 weeks post psychedelic use. Therefore, this study aimed to identify variables that are most strongly associated with study noncompletion, hoping to shed light on the extent of potential attrition biases in this and similar prospective studies on psychedelics.

Statistical analyses included multiple variables and were therefore exploratory; however, we were particularly interested in the effects of positive attitudes toward psychedelic use at baseline and of difficult drug experiences on attrition, owing to their potential implications for data interpretability. Specifically, we hypothesized that study completers would have higher baseline psychedelic advocacy scores and less challenging acute drug experiences than dropouts. Based on previous studies, we also hypothesized that dropouts would be younger and have lower educational levels, employment rates, and mental health than completers.

## Methods

### Ethics Approval

The study was approved by Imperial College Research Ethics Committee and the Joint Research Compliance Office at Imperial College London and carried out in accordance with principles of good clinical practice. Written informed consent was obtained from all subjects. The original survey is now closed, but the revised and still active versions of related surveys are available on the PsychedelicSurvey website [42].

### Design

Data were collected as part of a larger prospective study [9], approved by the Imperial College Research Ethics Committee and Joint Research Compliance Office. Only the elements of the design and data, which are relevant to this study, are presented here. Data were collected on the internet from a convenience sample of psychedelic drug users in a noncontrolled, naturalistic, and observational manner, through the website and software PsychedelicSurvey platform [42]. The open survey study was advertised on social media platforms, and informed consent was collected through a button-click feature following information on study purpose, design, and recruitment criteria. After sign-up, participants received emails that contained links to the relevant surveys at multiple timepoints, which were implemented and hosted by the web-based service system Alchemer [43]. Data were collected through a prospective cohort design: the baseline timepoint was set at 1 week before a planned psychedelic experience (timepoint 1), preacute measures were taken 1 day before the planned psychedelic experience (timepoint 2), and postacute measures were taken 1 day after the planned psychedelic experience (timepoint 3); the first endpoint was 2 weeks after the relevant experience (timepoint 4). The subsequent key endpoint occurred 4 weeks after the planned psychedelic experience (timepoint 5).

Baseline demographic and trait variables, postacute subjective effects measures, and outcome measures at the first endpoint were used to predict attrition. The survey 4 weeks after the psychedelic experience was the key endpoint, the completion of which was used as the criterion to determine attrition vs completion of the study. Dropouts were defined as those participants who stopped responding to the study surveys and did not return to finish the key endpoint, and completers were defined as those participants who reached the key endpoint 4 weeks after the psychedelic experience, even if they missed one or more previous timepoints.

### Measures

#### Baseline

The following measures were recorded at baseline: demographics (including age, gender, education, and employment status) and scores on the Warwick-Edinburgh Mental Well-being Scale (WEMWBS) [44] that is used to asses psychological well-being, the 10-Item Personality Inventory [45], the Social Connectedness Scale [46], a modiﬁed version of the Tellegen Absorption Scale [47] that is used to measure trait absorption and is a reliable predictor of the intensity of psychedelic experiences [9], the short version of the Spielberger State-Trait Anxiety Inventory (STAI-SF) [48], the 16-item Quick Inventory of Depressive Symptomatology (QIDS) [49], the Suicidal Ideation Attributes Scale (SIDAS) [50], and Peters’ Delusional Inventory (PDI) [51]. Additionally, 4 self-constructed items (“I am an active advocate of psychedelic drug-use,” “I am an active advocate of the therapeutic use of psychedelics,” “I have an advanced knowledge about psychedelics,” and “I am an highly experienced psychedelic user”) measured on a 5-point Likert scale were used to assess the advocacy of psychedelic drug use. A sum score was calculated on the basis of these 4 items, termed “psychedelic advocacy.” Cronbach α, as a measure of internal consistency among these 4 items, indicated acceptable internal consistency at α=.78 (bootstrap 95% CI 0.75-0.81 for 1000 samples).

#### Postacute Timepoint

Retrospective surveys pertaining to the acute experience were answered 1 day after the relevant psychedelic experience. These included a visual effects (VE) subscale of the 11-dimension altered states of consciousness questionnaire [52]; the Mystical Experience Questionnaire [53] that assesses acute positive peak experiences; the Challenging Experience Questionnaire [54] that is a measure of unpleasant affective, cognitive, and somatic difficulties experienced during psychedelic use; the Ego Dissolution Inventory [55] that addresses reductions in self-referential processing; the Emotional Breakthrough Inventory [11]; and the Physical Symptoms Inventory [56] that helps determine the number of several physical symptoms possibly experienced postacutely.

#### Endpoints

Outcomes were collected twice more during follow-ups 2 weeks and 4 weeks after the experience, including the scores on the WEMWBS, modiﬁed version of the Tellegen Absorption Scale, QIDS, STAI-SF, and PDI.

### Statistical Analysis

Logistic regression was used to assess the influence of the included variables on premature study termination, using the binary outcome attrition or completion as the dependent variable, where a participant was considered a completer if they had responded successfully to the key endpoint 4 weeks after the psychedelic experience. Owing to its nonreliance on the normal distribution of predictor variables, logistic regression is well-suited for the analysis of skewed data that have limited variability. Four models were fitted: 2 models containing baseline variables (1 including demographics, the other psychological measures), 1 containing postacute measures of psychedelic effects, and 1 with changes on relevant outcome variables between baseline and the 2-week endpoint. For those categorical and ordinal variables included in the demographic logit, we chose “Male” as reference category of gender owing to its greater size (n=485) compared to other levels of the variable, and the educational level “secondary education” owing to both its association with premature study termination known from the literature and its relevance in terms of sample size (n=276). Owing to a significant correlation (ρ=0.18; *P*<.001) and to reduce multicollinearity issues, it was decided to include only the educational level but not employment status as a predictor of attrition in the demographics logit. We also performed logistic regression analysis to investigate whether differences in psychedelic-induced changes on relevant outcome variables account for the likelihood of attrition. To compute change scores, individual baseline scores were subtracted from scores at the 2-week endpoint for each variable of interest (ie, WEMWBS, QIDS, STAI-SF, and PDI). Subsequently, a single logit was fitted, which included both change scores and absolute baseline scores, to account for the effect baseline variables. All statistical analyses were conducted using R (version 3.6.1, 2019-07-05; The R Foundation).

## Results

### Attrition Rates

**Figure 1:**
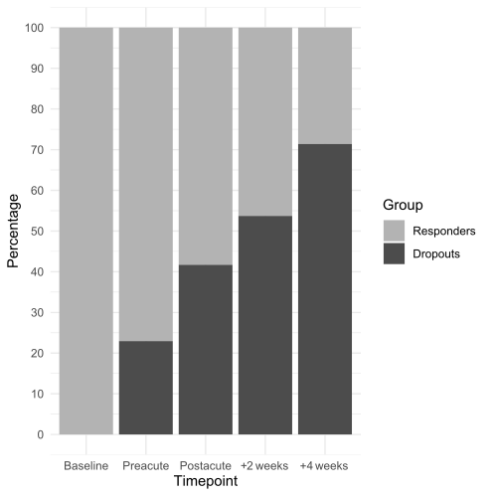
Cumulative frequency of participants dropping out (dark grey) and continuing to respond (light grey) to the key endpoint 4 weeks after a psychedelic experience among the sample sizes at each timepoint.

In total, 564, 535, 379, 315, and 212 participants were sampled at survey timepoints 1, 2, 3, 4, and 5, respectively. Since some participants did not complete the baseline survey but responded at a subsequent timepoint, the overall number of participants was >654, (n=741). Of this total of 741 participants responding at any study timepoint, 529 (71.4%) subsequently dropped out at or prior to the final survey at 4 weeks post psychedelic experience. In particular, 170 (22.9%) participants stopped responding 1 day prior to the planned psychedelic experience (preacute timepoint), 139 (18.8%) stopped 1 day post experience (postacute timepoint), 89 (12%) stopped at the 2-week endpoint, and 131 (17.7%) stopped at the second follow-up and key endpoint 4 weeks post experience (Figure 1).

The distribution of dropouts according to timepoint was significantly different (*χ*²_4_=56.89; *P*<.001); more noncompleters dropped out prior to rather than after the postacute timepoint: 309 (58.4%) vs 220 (41.6%), respectively (*χ*²_1_=29.28; *P*<.001). In total, 212 (28.6%) participants completed the second follow-up 4 weeks post experience and were thus classified as completers. Table 1 lists the demographics of participants at each timepoint of interest in this study.

**Table 1:** Demographics of participants at each time point.

Variables											Total		Dropouts		Completers
**Baseline**															
				Number of participants							654		469		185
				Age (years), mean (SD)							28.9 (10.4)		27.8 (9.9)		31.5 (11.5)
				**Gender, n (%)**											
					Male						485 (74.2)		359 (76.5)		126 (68.1)
					Female						165 (25.2)		107 (22.8)		58 (31.4)
					Other						4 (0.6)		3 (0.6)		1 (0.5)
				**Educational level, n (%)**											
					Primary education						53 (8.1)		34 (7.2)		19 (10.3)
					Secondary education						276 (42.2)		223 (47.6)		53 (28.7)
					University degree						325 (49.7)		212 (45.2)		113 (61.1)
				**Employment status, n (%)**											
					Unemployed					53 (8.1)		34 (7.3)		19 (10.3)	
					Student					256 (39.1)		198 (42.2)		58 (31.3)	
					Employed					335 (51.2)		232 (49.5)		103 (55.7)	
					Retired					10 (1.5)		5 (1.1)		5 (2.7)	
**Postacute timepoint (+1 day)**															
				Number of participants							379		192		187
				Age (years), mean (SD)							30.6 (11.0)		29.8 (10.5)		31.5 (11.5)
				**Gender, n (%)**											
					Male						252 (66.5)		138 (71.9)		114 (61)
					Female						97 (25.6)		42 (21.9)		55 (29.4)
					Other						2 (0.5)		1 (0.5)		1 (0.5)
					N/A^a^						28 (7.4)		11 (5.7)		17 (9.1)
				**Educational level, n (%)**											
					Primary education						30 (7.9)		12 (6.3)		18 (9.6)
					Secondary education						121 (31.9)		76 (39.6)		45 (24.1)
					University degree						200 (52.8)		93 (48.4)		107 (57.2)
					N/A						28 (7.4)		11 (5.7)		17 (9.1)
				**Employment status, n (%)**											
					Unemployed					33 (8.7)		15 (7.8)		18 (9.6)	
					Student					124 (32.7)		71 (37.0)		53 (28.3)	
					Employed					188 (49.6)		94 (49)		94 (50.3)	
					Retired					6 (1.6)		1 (0.5)		5 (21.7)	
					N/A					28 (7.4)		11 (5.7)		17 (9.1)	
**First endpoint (+2 weeks), n (%)**															
				Number of participants							315		131		184
				Age (years), mean (SD)							31.2 (11.2)		30.5 (10.7)		31.7 (11.6)
				**Gender, n (%)**											
					Male						196 (62.2)		82 (62.6)		114 (62.0)
					Female						82 (26.0)		32 (24.4)		50 (27.2)
					Other						1 (0.0)		0 (0.0)		1 (0.5)
					N/A						36 (11.4)		17 (13.0)		19 (10.3)
				**Educational level, n (%)**											
					Primary education						23 (7.3)		4 (3.1)		19 (10.3)
					Secondary education						87 (27.6)		43 (32.6)		44 (23.9)
					University degree						169 (53.7)		67 (51.1)		102 (55.4)
					N/A						36 (11.4)		17 (13)		19 (10.3)
				**Employment status, n (%)**											
					Unemployed					27 (8.6)		11 (8.4)		16 (8.7)	
					Student					86 (27.3)		38 (29.0)		48 (26.1)	
					Employed					159 (50.5)		63 (48.1)		96 (52.2)	
					Retired					7 (2.2)		2 (1.5)		5 (6.7)	
					N/A					36 (11.4)		17 (13)		19 (10.3)	

^a^Data at later timepoints were as not available for participants who had not completed the baseline questionnaire.

### Logistic Regression

Table 2 presents the results of logistic regression analysis. Age significantly predicted attrition (β=–0.024; *P*=.007); specifically, older age was associated with a reduced probability of dropping out from the study. Participants with a university degree were less likely to drop out than those with a secondary educational level (β=–0.574; *P*=.005), and participants with primary education were also less likely to drop out than those with secondary education (β=–0.876; *P*=.008). Personality trait extraversion significantly predicted attrition (β=0.082; *P*=.012): higher scores on extraversion were associated with an increased probability of dropping out from the study. Personality trait conscientiousness also significantly predicted attrition (β=–0.079; *P*=.024) in the opposite direction: higher scores on conscientiousness were associated with a reduced probability of dropping out. All other assessed variables included in logistic regression analyses were nonsignificant in predicting attrition, including any measures of the acute psychedelic state and measures potentially related to adverse events, such as suicidality, delusional thinking, physical side effects, or challenging experiences. Similarly, no psychedelic-induced changes on outcome variables significant predicted study attrition.

**Table 2:** Results of logistic regression models predicting noncompletion of the study key endpoint.

Variables							β^a^		SE		*z* value		*P* value
**Baseline demographics**													
			Intercept				1.633	0.509		3.209		.001	
			Age				–0.024	0.009		–2.684		*.007* ^b^	
		**Gender (reference: male)**											
					Female		–0.296	0.202		–1.460		.14	
					Other		–0.181	1.172		–0.155		.88	
		**Educational level (reference: secondary education)**											
				University degree			–0.574		0.206		2.786		*.005*
				Primary education			–0.876		0.329		–2.660		*.008*
				Psychedelic advocacy			0.031		0.027		1.124		.26
		**Psychological variables**											
				Intercept			2.355		1.293		1.822		.07
				Depression (QIDS^c^)			0.018		0.031		0.598		.55
				Well-being (WEMWBS^d^)			–0.011		0.017		–0.618		.54
		**Personality (TIPI)**											
						Extraversion	0.082		0.037		2.518		*.01*
						Conscientiousness	–0.079		0.035		–2.251		*.02*
						Agreeableness	–0.018		0.041		–0.446		.66
						Emotional stability	0.051		0.043		1.189		.23
						Openness to experience	–0.044		0.052		–0.848		.40
			Trait anxiety (STAI-SF^e^)				–0.016		0.012		–1.377		.17
			Social connectedness (SCS^f^)				–0.007		0.012		–0.646		.52
			Trait absorption (MODTAS^g^)				–0.000		0.007		–0.080		.94
			Suicidal ideation (SIDAS^h^)				0.013		0.016		0.831		.41
			Delusional thinking (PDI^i^)				0.035		0.029		1.196		.24
**Postacute timepoint (+1 day)**													
			Intercept				–0.257		0.317		–0.809		.42
			Visual effects				0.002		0.002		1.078		.28
			Challenging experience (CEQ^j^)				0.001		0.007		–0.117		.91
			Ego dissolution (EDI^k^)				–0.010		0.006		–1.535		.13
			Mystical experience (MEQ^l^)				0.012		0.009		1.414		.16
			Emotional breakthrough (EBI^m^)				–0.007		0.005		–1.409		.16
			Physical side-effects (PSI^n^)				–0.010		0.057		–0.181		.86
			Intercept for baseline–2-week changes				–1.590		2.312		–0.688		.49
**Change scores (baseline to +2 weeks)**													
		Depression (QIDS)					–0.014		0.062		–0.221		.83
		Psychological well-being (WEMWBS)					0.012		0.026		0.457		.65
		Trait anxiety (STAI-SF)					0.027		0.024		1.137		.26
		Social connectedness (SCS)					0.026		0.019		1.413		.16
		Delusional thinking (PDI)					0.034		0.060		0.565		.57
**Baseline control variables**													
	Depression (QIDS)						0.022		0.056		0.411		.68
	Psychological well-being (WEMWBS)						0.001		0.029		0.028		.98
	Trait anxiety (STAI-SF)						–0.014		0.021		–0.667		.51
	Social connectedness (SCS)						0.019		0.018		1.019		.31
	Delusional thinking (PDI)						0.044		0.039		1.144		.25

^a^β: estimated regression coefficient.

^b^Italicized values indicate significance levels of *P*<.05.

^c^QIDS: Quick Inventory of Depressive Symptomatology.

^d^WEMWBS: Warwick-Edinburgh Mental Well-being Scale.

^e^STAI-SF: Spielberger State-Trait Anxiety Inventory.

^f^SCS: Social Connectedness Scale.

^g^MODTAS: modiﬁed version of the Tellegen Absorption Scale.

^h^SIDAS: Suicidal Ideation Attributes Scale.

^i^PDI: Peters’ Delusional Inventory.

^j^CEQ: Challenging Experience Questionnaire.

^k^EDI: Ego Dissolution Inventory.

^l^MEQ: Mystical Experience Questionnaire.

^m^EBI: Emotional Breakthrough Inventory.

^n^PSI: Physical Symptoms Inventory.

## Discussion

### Principal Findings

This study aimed to identify variables accounting for attrition in prospective web-based studies on naturalistic psychedelic use. The overall attrition rate, which increased as a function of time, was high at 71.4% (n=529 of 741 participants). Contrary to prior hypotheses, neither the intensity of challenging experiences nor the advocacy of psychedelic use measured at baseline significantly predicted study completion. Rather, demographic variables including age and education, as well as personality traits including conscientiousness and extraversion, affected the likelihood of study noncompletion.

Specifically, logistic regression analyses revealed that young age, a low educational level, and the big 5 personality traits (high) extraversion and (low) conscientiousness were predictors of study attrition. The finding of age is in line with a number of previous studies [16-19] and may act synergistically with low conscientiousness to increase the likelihood of dropout. Indeed, several cross-sectional and longitudinal studies have shown that conscientiousness tends to increase with age [57-60], and that these increases often occur only during adulthood, which is relevant to the present study population, which had an average age of 28.9 (SD 10.4) years. The demographic logit also showed that participants with secondary education were more likely to drop out than those with university degrees and those with primary education. This may in part also be related to conscientiousness, which is known to be associated with educational expectations [61]. Highly conscientious young people also perform better academically and gain more advanced educational qualifications [62]. The personality trait conscientiousness is defined as “the propensity to follow socially prescribed norms and rules, to be goal-directed, planful, able to delay gratification, and to control impulses” [63]; thus, a conscientious person may indeed be more likely to commit to any obligation that he/she undertakes, be it loyalty to one’s partner, paying taxes, or completion of a survey pertaining to psychedelic drug use. The finding that extraversion was a significant predictor of study attrition is also in line with previous studies reporting that extraversion predicts premature termination in longitudinal studies [34,64,65].

The absence of influence from any of the psychedelic-specific predictors is an important finding. Several previous studies [9-11,41] have indicated the quality of the acute psychedelic state to be a reliable predictor of longer-term psychological changes following psychedelic use. Recognizing the importance of acute subjective drug effects has been a key consideration informing the renewed interest in the therapeutic value of psychedelic compounds [41,66]. In the present study, neither the quality of the acute experience nor psychedelic advocacy or psychedelic-induced long-term psychological changes predicted study attrition. Given the accumulating number of studies reporting improvements in mental health outcomes after naturalistic psychedelic use [9,67-73], as well as impactful clinical trials involving psychedelic interventions [3,74-81], it can be considered reassuring that none of the established mediators of positive outcomes, nor outcomes themselves, seem to bias study attrition in longitudinal studies on psychedelics. Although it is, by definition, impossible to address with certainty how the noncompleters in this study sample faired in terms of their postpsychedelic mental health, the absence of a relationship between attrition and biased perspectives toward psychedelics at baseline, the nature of the participants’ acute experiences, or the reported beneficial effects (ie, psychological changes), partly ameliorates previous concerns regarding attrition bias in observational studies on psychedelics [9,10,39].

From among 529 dropouts, 309 (58.4%) stopped responding prior to the postacute timepoint, and approximately one-third did so even prior to the preacute timepoint, which could potentially be explained by impaired accessibility to and reduced desire to access the survey directly prior to and after the psychedelic experience or by the postponement or nonperformance of the experience. Although surveys could be completed through the mobile phone, it is conceivable that a portion of participants who consumed the substance under field conditions (eg, at a festival) stopped responding after the baseline assessment owing to pragmatic reasons of limited access as well as decayed motivation. As highlighted by others [29,30,82], predictors of attrition often differ for early vs late dropouts, and such differences may be important factors that allow for targeted interventions to reduce attrition; for instance, by activating reminders on mobile devices in the early phases of the prospective study. The effects of the substance use environment, associated intentions, and other contextual factors of psychedelic use on attrition should be targeted in future studies.

### Limitations

Together, the observed effects suggest that the principal reasons for study attrition in observational studies on psychedelics are largely similar to those evidenced by other longitudinal studies that assessed phenomena independent of and unrelated to psychedelic use, with demographics and personality traits being the most predictive. Nevertheless, the present negative results with regard to potentially problematic systematic biases should only be considered preliminary, considering the limitations associated with the prevailing study design. Most significantly, the absence of data for those individuals who dropped out before completing the relevant surveys implies that both adverse experiences may have still occurred but were merely undetected. In future studies, with prior consent, this issue could be tackled by automatically sending out very brief surveys (eg, single-item surveys) to nonresponders, to investigate their reason for nonresponse. Similarly, rare cases of extreme negative reaction driving subsequent dropout may have been missed owing to both dropout and group averaging. Future studies focusing on such negative outliers may create pre-emptive value, given the disproportionate attention that can be attracted by such cases and the damaging impact this can have on broader studies and clinical development efforts. Further limitations of our study include a self-selection recruitment bias, which, as discussed by Haijen [9], reflects in a predominantly young, male, highly educated sample displaying strong psychedelic advocacy. On a more general level, the comparability of attrition studies across scientific domains and disciplines is a nontrivial problem. For example, outcome measures will not be consistent across studies, with the exception of simple demographic factors such as age. As revealed by independent studies [83-85], those on the predictors of attrition are often inconclusive, inconsistent, lacking in generalizability, and vulnerable to design-related limitations including a lack of standardization in definitions of attrition itself. Nevertheless, some evidence is convergent and our findings did generally converge consistently.

### Conclusions

This study sought to identify factors accounting for the high attrition rates in a prospective study on naturalistic psychedelic use. Consistent with findings from other scientific disciplines, the strongest predictors of study attrition were observed among variables including age, educational level, and personality traits. In contrast, psychedelic-specific factors were found to be poor predictors of attrition. Methods for reducing attrition, which have been validated through other fields, such as text messaging [86], gamification [87,88], monetary incentives [89,90], or the creation of web-based participant communities [91], are thus likely to be applicable also to observational studies on psychedelics, which should be investigated in future studies. While not without prevailing limitations, for which there is significant scope for improvement, these findings somewhat support the reliability and validity of large-scale prospective web-based data collection as a methodology for studying the predictors and processes of changes related to psychedelic use.

## Abbreviations

PDI: Peters’ Delusional Inventory
QIDS: Quick Inventory of Depressive Symptomatology
SIDAS: Suicidal Ideation Attributes Scale
STAI-SF: Spielberger State-Trait Anxiety Inventory
WEMWBS: Warwick-Edinburgh Mental Well-being Scale
